# Calibrated abdominal compression to assess fluid responsiveness in extremely and very preterm neonates: a pilot study

**DOI:** 10.3389/fped.2026.1711753

**Published:** 2026-04-09

**Authors:** Julien Gotchac, Noémie Jammet, Rémy Gérard, Damien Brousse, Justine Egger, Eloïse Le Cabec, Claire Renard, Olivier Tandonnet, Pascal Amedro, Sophie Cramaregeas

**Affiliations:** 1 Department of Pediatric and Congenital Cardiology, M3C National Reference Center, Bordeaux University Hospital, Pessac, France; 2 Liryc University Hospital Institute, INSERM 1045, University of Bordeaux, Bordeaux, France; 3 Department of Pediatric Intensive Care Unit, Bordeaux University Hospital, Bordeaux, France; 4 Department of Neonatal Intensive Care Unit, Bordeaux University Hospital, Bordeaux, France

**Keywords:** circulatory failure, echocardiography, fluid responsiveness, fluid therapy, preterm infants, shock, volume expansion

## Abstract

**Introduction:**

Predicting fluid-responsiveness is challenging in preterm neonates. It is however crucial to avoid unnecessary fluid bolus that could lead to fluid overload. In children, stroke volume changes induced by an abdominal compression (ΔSV-AC) can predict fluid responsiveness. This exploratory pilot study aimed to evaluate the feasibility and tolerance of this preload challenge in preterm neonates.

**Materials and methods:**

This prospective, single-center pilot study was conducted in a tertiary neonatal intensive care unit. Mechanically-ventilated and sedated preterm neonates under 32 weeks of corrected gestational age who required a 10 mL.kg^−1^ fluid bolus were eligible. Stroke volume was measured by echocardiography at baseline, during a gentle abdominal compression, and after the fluid bolus. A ≥15% stroke volume increase after fluid bolus defined fluid-responsiveness. In exploratory analysis, area under the receiver operating characteristic curve (AUROC) of ΔSV-AC was measured to predict fluid-responsiveness.

**Results:**

Eighteen fluid boluses were analyzed. Fluid-responsiveness was observed in 8 (44%) cases. The calibrated abdominal compression and the echocardiographic measurements were feasible in all cases. Although no serious adverse events were attributed to the maneuver, we observed three cases of transient but significant decreases in stroke volume or heart rate, two of which were accompanied by a subjective impression of poor tolerance. All other cases were subjectively rated as well tolerated. In exploratory analysis, after adjustment for repeated measures, the AUROC of ΔSV-AC to predict fluid-responsiveness was 0.76 (95% CI 0.43–1). The best threshold for ΔSV-AC was 17% with a specificity of 0.91 (95% CI 0.60–1), a sensitivity of 0.51 (95% CI 0.17–1), and positive and negative predictive values of 0.85 (95% CI 0.36–1) and 0.68 (95% CI 0.33–1) respectively.

**Conclusions:**

This study suggests that calibrated abdominal compression could be feasible in a population of critically ill preterm neonates mostly suffering from PPHN-related shock, although its tolerance is uncertain. Further studies are needed to better tailor this maneuver to preterm neonates and to characterize its diagnostic accuracy, including in more common etiologies of neonatal shock.

**Clinical Trial Registration:** https://clinicaltrials.gov/study/NCT06287710, identifier NCT06287710.

## 1 Introduction

A fluid bolus (FB) is often the initial intervention to treat hypotension in extremely preterm neonates (1–3). Although there is no clear correlation between blood volume and blood pressure in neonates, FB can increase stroke volume (SV) and cardiac output in fluid responsive patients (4). However, in fluid unresponsive patients, FB may either be useless or harmful. Indeed, fluid restriction could be associated with decreased risk of patent ductus arteriosus, necrotizing enterocolitis and mortality (5–7). Furthermore, early fluid overload in extremely low birth weight infants is associated with higher mortality and respiratory morbidity (8, 9). This argues for a careful use of FB in preterm infants. However, although fluid responsiveness assessment has become a standard of care in pediatric and adult intensive care, the literature is still very limited for preterm infants (10).

In older children, the respiratory variability of the peak aortic velocity (ΔVPeak) is considered the best-validated fluid responsiveness test (11, 12). However, it has been little investigated in preterm newborns (13). Moreover, this test is based on cardiopulmonary interactions. Its use it therefore limited in children ventilated with high-frequency oscillation, as small tidal volumes are insufficient to induce a preload variation, and is those with right ventricular dysfunction in whom ventilation-induced afterload variations can induce SV variability regardless of volume status (11, 14, 15). Only dynamic tests based on another source of preload variation could be applied to these patients. In adults, the classic passive leg-raising test relies on an endogenous, reversible, and ventilation-independent increase in preload through the mobilization of the venous reservoir of the lower limbs (16). However, its diagnostic accuracy appears to be poorer in children (17–19). This might be due to a lower blood volume in the lower limbs, as leg length is proportionately smaller in infants (20). Conversely, the hepatosplanchnic venous reservoir is easily accessible in children. A gentle abdominal compression can rapidly mobilize the unstressed venous blood volume from the abdominal organs, transiently increasing venous return and cardiac preload (21). Previous reports have successfully used this technique to assess fluid-responsiveness in postoperative pediatric cardiac surgery (21–23) and in infants hospitalized in a general pediatric intensive care unit (24). However, these results cannot be extrapolated to preterm neonates due to differences regarding abdominal compliance and relationship between preload and stroke volume.

To date, the calibrated abdominal compression maneuver has never been evaluated in preterm neonates. We hypothesized that the measure of SV changes induced by a calibrated abdominal compression maneuver (ΔSV-AC) is feasible in mechanically ventilated extremely and very preterm neonates. Accordingly, this pilot study was designed primarily to assess feasibility and tolerance, with secondary exploratory and hypothesis-generating analyses focusing on signal detection regarding its potential association with fluid responsiveness.

## 2 Materials and methods

### 2.1 Study design, setting and population

This pilot study was prospectively conducted in a single tertiary neonatal intensive care unit in Bordeaux University Hospital, France, from February to July 2024. Mechanically ventilated and sedated preterm neonates under 32 weeks of corrected gestational age requiring FB (10 mL.kg^−1^ I.V., over 20 min) were consecutively screened. As written in the bedside case report form, FB prescription was left to the attending physicians' own judgment, based on clinical, biological, or ultrasound data such as: heart rate (HR) > 180 min^−1^, arterial pressure bellow 3rd percentile, urine output < 1 mL.kg^−1^.h^−1^, capillary refill time > 3 s, mottling, blood lactate > 2 mmol.L^−1^, or low cardiac output. Exclusion criteria were congenital heart disease, cardiogenic pulmonary oedema, abdominal pain (defined as an increase in COMFORT-B scale during a previous abdominal palpation in a routine physical examination), necrotizing enterocolitis, postoperative period of abdominal surgery, prone position, imminent cardiac arrest due to circulatory failure requiring fluid bolus without any delay, poor ultrasound window and investigator unavailability. As per local protocols, all patients were sedated with a combination of Dexmedetomidine (or Midazolam as a second-line agent) and Sufentanil.

### 2.2 Experimental protocol

The calibrated abdominal compression maneuver was performed following a previously described standardized protocol (21, 24). A closed sphygmomanometer inflated with 50 mL of air was connected to a pressure-measuring device and interposed between the operator's hand and the patient's abdomen. The sphygmomanometer was placed at the center of the patient's abdomen to cover 30 to 60% of its surface. The operator then performed a gentle manual compression in an anteroposterior direction, gradually reaching a pressure of 22 mmHg for 30 s (Supplementary Figure S1, Supplementary Material). This fixed compression pressure was selected as a pragmatic compromise to balance feasibility, external validity and patient safety. Before choosing it, experienced neonatologists were asked to blindly simulate a gentle abdominal palpation of an unstable preterm newborn. As the pressure found was greater than 22 mmHg in all cases, this compression pressure was favored because it was already validated in full-term newborns (21, 24). Tolerance was assessed by hemodynamic variations during the maneuver (increase or decrease ≥10% in HR, or decrease ≥10% in cardiac index), by the need for resuscitation following the maneuver, and by the subjective perception of the physician in charge and of the operator performing the maneuver, if different. Although it was performed by a different operator than the one performing echocardiograms, it was not possible to perform the abdominal compression blinded to the measurements.

For each FB included, three series of echocardiographic measures were performed, at baseline (T0), after 30 s of the calibrated abdominal compression maneuver (T1), and 30 min after FB (T2). Left ventricular ejection fraction and left ventricular outflow tract (LVOT) diameter were measured at baseline. At each time point, 5 consecutive aortic velocity-time integrals (VTI) and mean aortic flow velocity (MAFV) were acquired from an apical five-chambers view, using pulse wave Doppler in the LVOT with the minimum possible insonation angle. Transthoracic echocardiograms were performed by a senior neonatologist with high experience in neonatal echocardiography (DB, OT or SC), using the Vivid S60 ultrasound system and a 12S probe (General Electric Medical Systems SCS, Boston, MA, USA). A second offline analysis was performed to assess interobserver reproducibility of VTI and MAFV measures. Although echocardiographic acquisition could not be blinded to clinical status, this second offline analysis was performed by a single investigator (NJ) which was not implied in echocardiographic bedside acquisitions, and without access to patient data. Offline measurements were made sequentially (T0 of all patients, then T1, then T2) to minimize potential bias. These offline values were used for primary analysis.

SV was calculated as $VTI\;\times \;\Pi \;\times \frac{LVOT\;d^{2}}{4}$. Cardiac index was the product of SV and HR, indexed by body surface area. The change in SV during the calibrated abdominal compression, e.g., ΔSV-AC (%), was measured by the difference between SV at T1 and SV at T0, divided by SV at T0 (index test). The change in SV after FB, e.g., ΔSV-FB (%) was measured by the difference between SV at T2 and SV at T0, divided by SV at T0. Fluid responsiveness was defined by an increase in ΔSV-FB ≥ 15%, as it is the most used reference standard fluid responsiveness test in pediatrics (14, 15, 25, 26), and has also been previously used in preterm neonates (27). Although this threshold might seem arbitrary and does not reflect the continuous nature of fluid responsiveness, it is usually chosen because it matches the lowest change in cardiac output that transthoracic echocardiography can reliably detect (28). However, this has not been formally evaluated in extremely and very preterm neonates.

At baseline, the presumed origin of shock according to the physician in charge was collected: cardiogenic, hypovolemic, septic, related to persistent pulmonary hypertension of the newborn (PPHN), or other cause. PPHN was defined as hypoxemia with a pre- and postductal SpO₂ difference greater than 5%, related to a right-to-left shunt through the ductus arteriosus, and associated with the following echocardiographic abnormalities: flattening or leftward deviation of the interventricular septum, increased tricuspid regurgitation velocity assessed by continuous-wave Doppler, right ventricular dilation and/or dysfunction (29, 30). Shock was assumed to be related to PPHN if case of associated low cardiac output and without evidence for other etiologies. Clinical and hemodynamic parameters were also collected at baseline and after FB, including patient characteristics, ventilation mode, fraction of inspired oxygen, mean airway pressure, presence of a vasoactive or inotropic hemodynamic support, vasoactive-inotropic score, HR, noninvasive mean systemic arterial pressure, mottling, capillary refill time, urine output, peripheral oxygen saturation, and blood lactate if available. Duration of mechanical ventilation and mortality were collected at discharge. Finally, the perception of the neonatologist in charge of the patient regarding FB efficacy was collected immediately before the post-FB echocardiographic assessment at T2.

### 2.3 Statistical analysis

Due to the exploratory nature of this pilot study, no formal sample size or power calculation was performed. All patients who met the eligibility criteria within a pre-established six-month period were included. Characteristics were presented in median form [interquartile range], or as frequencies and proportions for qualitative variables.

For categorical variables, group comparisons were performed with an exact Fisher test. Quantitative variables were compared using the Mann–Whitney test. Spearman correlation coefficient was used to test linear correlations. The reproducibility of VTI and MAFV measures within each acquisition was evaluated by calculating intraclass correlation coefficients to assess interobserver reliability. The Wilcoxon test was used to compare parameters between before and after FB. For secondary exploratory analysis, receiver operating characteristic (ROC) curves were drawn to determine the ability of ΔSV-AC to predict fluid responsiveness. The area under the ROC curve (AUROC), with its 95% confidence interval (95% CI) represented the overall diagnostic accuracy of ΔSV-AC. The best ΔSV-AC threshold value was determined using the Youden method. Sensitivity, specificity, positive and negative predictive values were identified at this threshold value, with their respective 95%CI. To account for repeated measures related to the possible inclusion of multiple FB per patient, a clustered bootstrap resampling at patient level (1000 iterations) was performed. A binomial logistic regression was used to identify parameters associated with fluid responsiveness. The two groups, e.g., fluid responsive vs. unresponsive cases, were analyzed according to FB cases and not to patients, as some patients underwent multiple FB. Since repeated measurements obtained from the same patients might be correlated, a binomial logistic regression with generalized estimating equations was also performed. Analyses were performed with RStudio version 4.0.3 (RStudio, PBC, Boston, MA, USA) and the Jamovi 1.2 graphical interface (The Jamovi project, Sydney, Australia). Differences with a P-value of less than 0.05 were considered statistically significant.

### 2.4 Ethics

The study was carried out in accordance with the Good Clinical Practices protocol and Declaration of Helsinki principles. It was approved by an Institutional Review Board (2023-A02013-42) and registered on Clinicaltrials.gov (NCT06287710, January 18, 2024). Informed oral consent was obtained from both parents or legal guardians for all participants, before or within 24 h after study procedures.

## 3 Results

**Figure 1:**
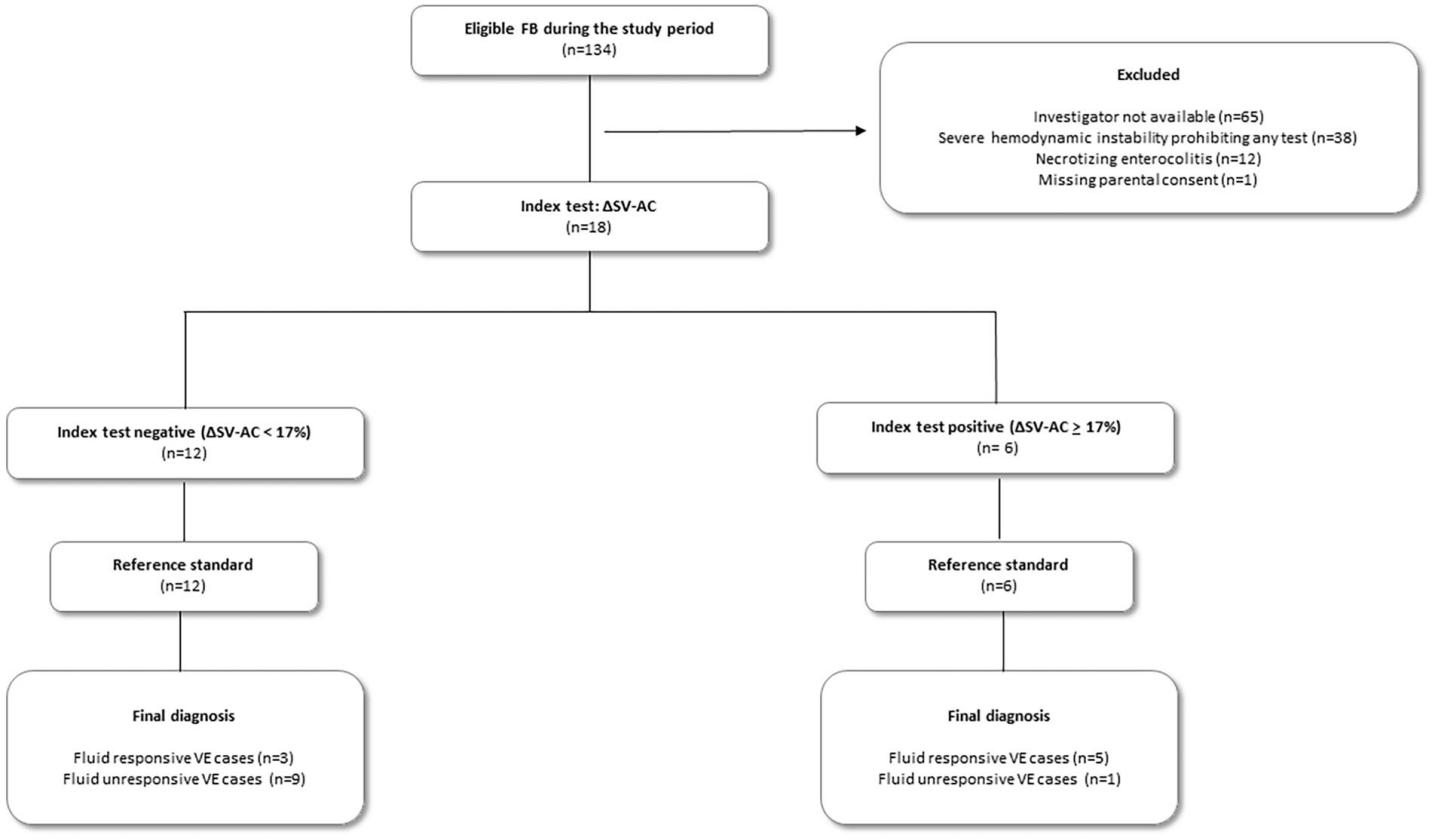
Study flow chart. ΔSV-AC, percentage of stroke volume variation between baseline and during calibrated abdominal compression, FB fluid bolus.

From February 2024 to July 2024, 18 cases of FB were prospectively collected from a cohort of 10 preterm neonates. The study flow chart is reported in Figure 1. Baseline characteristics of the 10 patients are reported in Table 1. Gestational age at birth ranged from 23.1 to 29.1 weeks. Among the 18 FB cases, median postnatal age at inclusion was 8 days [6;10], with a median corrected gestational age of 27.2 [25.6;28.4] weeks. The neonatologist in charge of the patient considered the shock to be related to persistent pulmonary hypertension of the newborn (PPHN) in 14 cases (78%), septic in 1 case (6%), and of another origin in 3 cases (17%): sedation-induce vasodilatory state (1 case) and systemic hypoperfusion due to shunt imbalance (2 cases). No patient had any invasive pressure measurement, as per the usual practice of this neonatal intensive care unit. Fluid used for volume expansion was 10% Albumin in 13/18 cases (72%), and balanced crystalloid in 5/18 cases (28%). Baseline clinical, biological, and echocardiographic data in both responsive and non-responsive FB-cases are reported in Table 2. After FB, 8/18 cases (44%) were classified as fluid-responsive (i.e., ΔSV-FB > 15%), while the neonatologist in charge perceived improvement in 15/18 cases (83%). Hemodynamic and respiratory changes after FB are reported in Table 3.

**Table 1:** Population demographic and clinical characteristics.

Variable	Patients (*N* *=* *10*)
Birth gestational age (weeks)	25.5 [24.3;26.4]
Male (*N*, %)	8 (80%)
IUGR (*N*, %)	5 (50%)
NICU mortality (*N*, %)	6 (60%)

IUGR, intra uterine growth restriction; MV, mechanical ventilation; NICU, neonatal intensive care unit. Data are reported as No. (%) or medians [first quartile; third quartile].

**Table 2:** Baseline group characteristics.

Variable	All FB cases (*N* = 18)	Fluid-unresponsive cases (*N* = 10)	Fluid-responsive cases (*N* = 8)	*p* value
Male sex (%)	14 (78%)	9 (90%)	5 (63%)	0.275
Weight (g)	770 [690;890]	730 [690;940]	770 [710;840]	0.965
Birth gestational age (weeks)	25.4 [25.3;26.4]	25.3 [24.3;25.6]	26.7 [25.3;27]	0.066
Adjusted gestational age (weeks)	27.2 [25.6;28.4]	26.3 [24.8;28.8]	28.0 [26.4;28,3]	0.468
HFO ventilation (*N*, %)	17 (94%)	10 (100%)	7 (88%)	0.444
MAP (cmH_2_O)	16 [15;16]	16 [15.3;16.8]	16 [14.8;16]	0.514
FiO_2_ (%)	76 [57;100]	70 [57;99]	91 [57;100]	0.680
SpO_2_/FiO_2_ ratio (%)	115 [89;155]	126 [89;155]	103 [89;162]	0.929
OSI	14.0 [10.2;19.3]	12.7 [10;2;19.3]	15.6 [10.1;18.8]	0.904
Albumin FB (%)	13 (72%)	7 (70%)	6 (75%)	1.000
PPHN (*N*, %)	14 (78%)	7 (70%)	7 (88%)	0.588
Hemodynamic support (*N*, %)	17 (94%)	9 (90%)	8 (100%)	1.000
VIS (μg.kg^−1^.min^−1^)	28.2 [10.7;54.8]	28.3 [11.9;42.5]	26.6 [14.9;100.7]	0.894
Heart rate (beats.min^−1^)	170 [161;184]	165 [158;179]	175 [162;190]	0.266
Mean arterial pressure (mmHg)	40 [33;55]	36 [31;54]	42 [38;56]	0.315
Capillary refill time (sec)	2.0 [1.0;2.0]	1.5 [1.0;2.0]	2.0 [1.8;2.0]	0.225
Mottling	2 (11%)	2 (20%)	0 (0%)	0.477
Urine output (mL.kg^−1^.h^−1^)	4.5 [2.9;7.1]	3.5 [2.7;5.8]	5.8 [3.9;8.3]	0.159
Lactate (mmol.L^−1^)	2.8 [1.9;4.3]	2.6 [1.6;4.8]	2.8 [2.6;3.5]	0.790
LVEF (%)	80 [73;83]	82 [78;87]	76 [72;80]	0.120
**VTI (cm)**	**6.9 [6.3;8.5]**	**8.3 [6.7;9.7]**	**6.3 [4.4;6.8]**	**0**.**011**
MAFV (m.s^−1^)	0.23 [0.19;0.29]	0.28 [0.20;0.32]	0.21 [0.16;0.24]	0.068
SV (mL.m^−2^)	10.2 [7.1;11.9]	10.6 [8.8;14.6]	7.4 [6.0;10.7]	0.083
Cardiac index (mL.kg^−1^.min^−1^)	231 [181;263]	236 [231;283]	189 [150;231]	0.055

FiO_2,_ fraction of inspired oxygen; LVEF, left ventricular ejection fraction; MAFV, mean aortic flow velocity; MAP, mean airway pressure; HFO, high frequency oscillation; OSI, oxygen saturation index = mean airway pressure (cmH2O) × FiO_2_/SpO_2_; SpO_2_, peripheral oxygen saturation; PPHN, persistent pulmonary hypertension of the newborn; SV, stroke volume; VIS, vasoactive inotropic score; VTI, aortic velocity-time integral. Data are reported as No. (%), or medians [first quartile; third quartile]. Significant *p* values are marked in bold.

**Table 3:** Hemodynamic changes before and after fluid bolus .

Variable	Volume expansion		
	Before	After	*p* value
SpO2/FiO2 ratio (%)	115 [89;155]	144 [106;200]	0.009
Heart rate (min^−1^)	170 [161;184]	170 [157;177]	0.660
Mean arterial pressure (mmHg)	40 [33;55]	51 [44;63]	0.012
Capillary refill time (sec)	2.0 [1.0;2.0]	1.0 [1.0;1.0]	0.015
Urine output (mL.kg^−1^.h^−1^)	4.5 [2.9;7.1]	8.9 [5.8;10.0]	0.004
Lactate (mmol.L^−1^)	2.8 [1.9;4.3]	2.8 [2.2;4.7]	0.889
VTI (cm)	6.9 [6.3;8.5]	8.7 [6.6;9.8]	0.009
MAFV (m.s^−1^)	0.23 [0.19;0.29]	0.27 [0.21;0.31]	0.113
Cardiac index (mL.kg^−1^.min^−1^)	231 [181;263]	263 [216;326]	0.006

FiO_2,_ fraction of inspired oxygen; MAFV, mean aortic flow velocity; SpO_2_, peripheral oxygen saturation; VTI, aortic velocity-time integral. Data are reported as medians [first quartile; third quartile].

The calibrated abdominal compression and the echocardiographic measurements were feasible in all cases. The intraclass correlation coefficients for interobserver reliability was 0.95 for VTI and 0.89 for MAFV. Regarding tolerance of the calibrated abdominal compression, there was no significant HR variation during the maneuver compared to baseline (173 [164;182] vs 170 [161;184] bpm, *p* = 0.342). A significant decrease in cardiac index during the maneuver was observed in 2 cases, 1 of which was subjectively rated as poorly tolerated. A significant decrease in HR without decrease in cardiac index during the maneuver was observed in 1 case, which was also subjectively rated as poorly tolerated. However, no resuscitation maneuver was necessary, and no serious adverse events were directly attributable to the calibrated abdominal compression maneuver itself. Moreover, no objective clinical deterioration after FB was observed in any of these cases. All other cases were subjectively rated as well tolerated. Detailed tolerance data are reported in the Supplementary Material (Supplementary Table S1).

**Figure 2:**
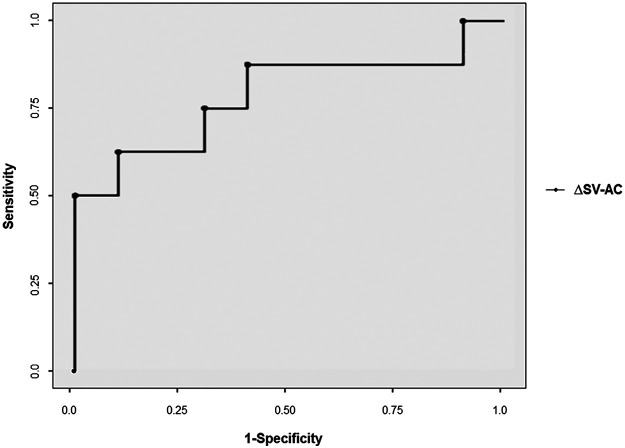
Roc curve of ΔSV-AC for predicting fluid responsiveness. ΔSV-AC, percentage of stroke volume variation between baseline and during calibrated abdominal compression.

The estimated ROC curve of ΔSV-AC is shown in Figure 2. The estimated AUROC of ΔSV-AC to predict fluid responsiveness was 0.79 (95% CI 0.55–1). Using the Youden method, the best threshold for ΔSV-AC was 17% with a specificity of 0.90 (95% CI 0.55–1), a sensitivity of 0.63 (95% CI 0.24–0.91), a positive predictive value of 0.83 (95% CI 0.36–1), and a negative predictive value of 0.75 (95% CI 0.43–0.95). The diagnostic performance after adjustment for repeated measures was: AUROC 0.76 (95% CI 0.43–1), best threshold 17%, specificity 0.91 (95% CI 0.60–1), sensitivity 0.51 (95% CI 0.17–1), positive predictive value 0.85 (95% CI 0.36–1), and negative predictive value 0.68 (95% CI 0.33–1). ΔSV-AC was significantly associated with ΔSV-FB (Pearson coefficient *r* = 0.601, *p* = 0.008), even when adjusted for patient weight (*r* = 0.604, *p* = 0.013) and for repeated measures (*r* = 0.71, *p* = 0.002). Sensitivity analyses were performed to account for potential biases induced by the heterogeneity of the population (e.g., ventilation, shock mechanism, fluid type and anteriority of FB), with similar results. Similar results were also observed using other definitions of fluid responsiveness. Sensitivity analyses are reported in the Supplementary Material (Supplementary Tables S2, S3).

In univariate analysis (Supplementary Table S4, Supplementary Material), only the baseline VTI was significantly associated with fluid responsiveness (OR = 0.43, 95% CI 0.19–0.99, *p* = 0.047). This association remained significant after adjustment for patient weight (OR = 0.35, 95% CI 0.14–0.93, *p* = 0.034) and after adjustment for repeated measurements within patients (OR = 0.42, 95% CI 0.25–0.68, *p* < 0.001). The univariate analysis adjusted for repeated measurements within patients (Supplementary Table S5, Supplementary Material) did not substantially impact the overall interpretation of these results.

## 4 Discussion

This pilot study reported that the calibrated compression maneuver was feasible in critically ill extremely and very preterm neonates, although tolerance data are equivocal. However, the wide confidence intervals due to the exploratory nature of this low-sample-size study precludes any firm conclusions regarding diagnostic performance.

In this study, most patients were extremely preterm infants suffering from PPHN-related shock, with severe respiratory and hemodynamic compromise. Although our population was very homogenous, this limits the extrapolation of our findings to more common etiologies of neonatal shock (such as septic or vasodilatory states). This understudied population is however of interest because the hemodynamic management of these patients is particularly challenging. Indeed, in the context of PPHN, the mechanism of hypotension is not always hypovolemia and unnecessary FB might exacerbate a right ventricular failure (31). Furthermore, avoiding fluid overload may be considered highly relevant for these fragile patients. In a study including 98 late preterm neonates with PPHN, the amount of FB during the first 7 days of hospitalization was associated with extracorporeal membrane oxygenation requirement or death (32). As observed in older children and adults (15, 33), only 44% of FB were fluid responsive in our study. This highlights the importance of developing fluid-responsiveness tests tailored for this population, that could avoid unnecessary and potentially harmful FB.

The abdominal compression maneuver and the echocardiographic assessment of changes in SV during the maneuver were feasible in all cases. This suggest that fluid responsiveness could possibly be predicted using a preload challenge in this fragile population. However, the calibrated abdominal compression maneuver in its current form might not be the perfect one in this population, for several reasons. Firstly, although no serious adverse events were attributed to the maneuver, tolerance data are equivocal. Indeed, we observed three cases of transient but significant decreases in stroke volume or HR, two of which were also subjectively rated as poorly tolerated. In all these cases, patients suffered from PPHN-related shock. However, no clinical deterioration in oxygenation between baseline and after FB was observed in these patients. All other cases had a good tolerance profile, although it has only been assessed by subjective perception and variations in HR and cardiac index. Theoretically, it is possible that the maneuver might secondarily impair venous return, through a compression of the inferior vena cava, and increase pulmonary vascular resistance, through sympathetic stimulation. These mechanisms might lead to a transient hemodynamic destabilization in the most unstable patients, especially patients already hypovolemic or suffering from PPHN. This advocates for more advanced hemodynamic and nociception monitoring in future similar studies. Secondly, a uniform compression pressure of 22 mmHg might not be appropriate, given wide variability in abdominal compliance, gestational age, and weight in preterm neonates. An ideal preload challenge should be individualized to increase mean systemic pressure in the same proportions in all patients (34). However, this is difficult to measure in practice. To individualize the compression pressure, it may be interesting to investigate in future studies whether anthropometric parameters predictably influence abdominal compliance and thus affect the relationship between an external compression pressure and the resulting increase in intra-abdominal pressure, monitored using a urinary catheter.

Due to the pilot nature of this study and the wide confidence intervals resulting from the low sample size, secondary results about diagnostic accuracy are difficult to interpret and compare with the existing literature. Indeed, to our knowledge, this study investigated for the first time the calibrated abdominal compression in premature neonates. Only one other study has investigated a preload challenge in this population, and the pilot data suggested that a mini-fluid challenge (3 mL.kg^−1^ over 5 min) to predict fluid responsiveness was feasible in the neonatal intensive care unit (27). However, our preliminary results seem consistent with studies on calibrated abdominal compression conducted in older children. Jacquet-Lagreze et al. found an AUROC of ΔSV-AC of 0.94 in 39 postoperative cardiac surgery children with a median age of 9 months (21). Our team found similar results in a previous study conducted in a general pediatric intensive care unit, in infants (mostly neonates) without cardiac disease (24).

Among baseline parameters, only VTI was significantly associated with fluid responsiveness, even after adjustment for repeated measurements within patients. Interestingly, MAFV and cardiac index were not. Regarding cardiac index, this could be explained by a higher variability of LVOT measurement, any errors in diameter being squared in the surface calculation. MAFV is a measure correlated with cardiac output, and has the theoretical advantage to be less dependent on HR than VTI (35). However, this parameter could be less reproducible than VTI and therefore exposed to greater measurement error. Moreover, the patients in this study all had relatively similar HR. Overall, our results support the use of simple and reproducible echocardiographic measures such as VTI when assessing the hemodynamics of these patients (36), although the reproducibility related to the acquirement of VTI at bedside was not evaluated in this study. Conversely, clinical parameters alone seem to be insufficient to guide the prescription of FB as previously reported (10), although this study was underpowered to properly address this issue.

This study has several limitations. First, although the population was quite homogeneous, the number of FB cases included was very small, reflecting the exploratory nature of this pilot study. This induced a lack of statistical power and wide confidence intervals, precluding any firm conclusion regarding secondary objectives such as diagnostic accuracy, parameters associated with fluid responsiveness, sensitivity analysis and subgroup analysis. These elements should therefore be regarded as hypothesis-generating only. Moreover, some patients were included multiple times if they received more than one FB, as the primary analysis was conducted at the FB-level. The rationale was that subjects with prior FB would have lower odds of being fluid-responsive than for their first FB. Therefore, had we included only the first FB of each patient, we would not have been able to fully capture the target population. However, as intrinsic patient factors could have influenced both ΔSV-AC and fluid responsiveness, those repeated measures are likely to be correlated and might violate the assumption of independence. Without adjustment for within-patient clustering, this may result in underestimation of variance and overestimation of diagnostic performance. However, analyses with approaches accounting for repeated measures and clustered ROC analysis found relatively similar results. Nevertheless, even these adjusted results should be interpreted cautiously, as the small number of clusters (*n* = 10 patients) may result in imprecise variance estimates. Second, some local practices do not match the usual current standards, especially the choice of fluid (including frequent use of albumin) and the absence of invasive arterial pressure monitoring. This further limits the interpretation and generalizability of our findings, already restricted to patients with PPHN, as discussed above. However, the non-interventional design of this study prohibited any modification of local protocols or the collection of any data that was not already being measured as part of patient care. Third, only patients for whom the clinician had already decided to prescribe a FB, based on their own judgment, were included. This may result in spectrum bias, as this approach might have enriched the study population with infants perceived by the physician as having a high probability of being fluid responder, for various possible reasons. Conversely, incidental findings during the echocardiography performed as part of fluid responsiveness assessment may have discouraged FB prescription for some patients. Consequently, the observed fluid responsiveness rate and the estimated diagnostic performance of ΔSV-AC might differ from those observed in a broader population of hypotensive neonates. However, administering fluid boluses to patients without clinical indication would not have been ethically acceptable in this non-interventional study. Moreover, fluid-responsiveness tests are, by design, intended for clinical situations in which FB administration is already being considered. Thus, this selected population closely matches the intended clinical target population. Fourth, the availability of an investigator limited the number of patients included, although this unlikely resulted in systematic selection bias. Finally, fluid responsiveness was assessed using echocardiography, i.e., an operator-dependent examination. Nevertheless, echocardiography is widely used as a gold standard pediatric test (14, 15, 26). Overall, while the main objective of this pilot study was the feasibility of a preload challenge maneuver in a real-life setting, all secondary analysis should be interpreted cautiously and regarded as hypothesis-generating only.

Overall, fluid responsiveness assessment seems to be particularly challenging in preterm neonates. In older children, the most widely studied and used tests are ΔVPeak and inferior vena cava respiratory variability (11, 12, 26). Recently, ΔVpeak has also been investigated in hemodynamically unstable ventilated neonates with promising results, but patients with PPHN, right heart failure or on high-frequency oscillatory ventilation were excluded (13). In our study, most patients had PPHN-related shock and were on high-frequency oscillatory ventilation, which can have significant hemodynamic effects in neonates (37, 38). For these reasons, all tests based on cardiopulmonary interactions are likely to be biased and unusable. Yet it seems particularly important to evaluate the benefit/risk ratio of FB in these fragile patients. There is a need for fluid responsiveness tests tailored for this population, i.e., based on a dynamic preload challenge rather than on cardiopulmonary interactions, and that require minimal contact to the patient.

## 5 Conclusion

This study suggests that calibrated abdominal compression could be feasible in a population of critically ill preterm neonates mostly suffering from PPHN-related shock, although its tolerance is uncertain. Further studies are needed to better tailor this maneuver to preterm neonates and to characterize its diagnostic accuracy, including in more common etiologies of neonatal shock. Indeed, there is an urgent need to intensify the clinical research on this topic and to develop fluid responsiveness tests that could prevent unnecessary FB in this vulnerable population.

## Data Availability

The raw data supporting the conclusions of this article will be made available by the authors, without undue reservation.
