# Supplementary material for: Calibrated abdominal compression to assess fluid responsiveness in extremely and very preterm neonates: a pilot study
Source: Front Pediatr. 2026 Apr 9;14:1711753. doi: 10.3389/fped.2026.1711753 (PMC13102776; doi:10.3389/fped.2026.1711753)
Supplement: Supplementary file 1 [file Supplementaryfile1.docx]

Calibrated abdominal compression to assess fluid responsiveness in extremely and very preterm neonates: a diagnostic accuracy study.

Supplementary Material

# Supplementary Figures and Tables

## Supplementary Figures

**
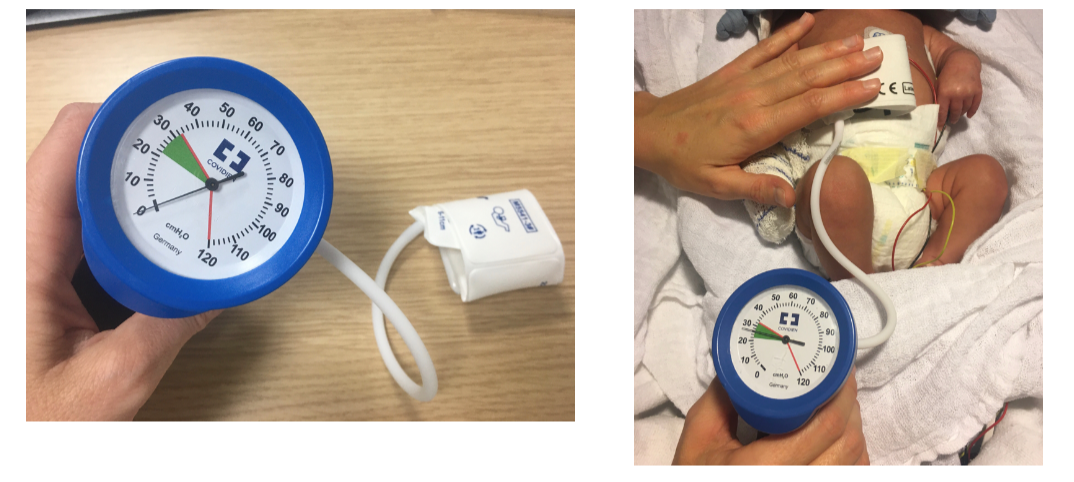
**

**Supplementary Figure 1. Practical example of the calibrated abdominal compression.** In this schematical illustration, the sphygmomanometer is previously inflated with 50ml of air. A manual anteroposterior abdominal compression is applied and calibrated at 22mmHg (=30 cmH2O) using this closed sphygmomanometer (connected to a pressure measuring device) interposed between the operator’s hand and the center of the patient’s abdomen.

## Supplementary Tables

### Supplementary Table 1. Tolerance Data.

| **Case n°** | **Significant HR variation during CAC** | **Significant CI decrease during CAC** | **Resuscitation following CAC** | **Physician's subjective perception** | **Shock mechanism** | **Patient outcome** | **Clinical deterioration after VE (MAP, lactate, oxygenation)** |
| --- | --- | --- | --- | --- | --- | --- | --- |
| 1 | No | No | No | Good tolerance | Other | Alive at discharge | No |
| 2 | No | Yes (-50%) | No | Poor tolerance | PPHN | Alive at discharge | No |
| 3 | No | No | No | Good tolerance | PPHN | Alive at discharge | No |
| 4 | No | No | No | Good tolerance | PPHN | Alive at discharge | No |
| 5 | No | No | No | Good tolerance | PPHN | Alive at discharge | No |
| 6 | No | No | No | Good tolerance | PPHN | Alive at discharge | No |
| 7 | Yes (from 200 to 180 bpm) | No (5% increase despite decrease in HR) | No | Poor tolerance | PPHN | Deceased | Lactate increase, normal MAP, oxygenation unchanged |
| 8 | No | No | No | Good tolerance | PPHN | Deceased | Decrease in oxygenation |
| 9 | No | No | No | Good tolerance | PPHN | Deceased | No |
| 10 | No | No | No | Good tolerance | Other | Alive at discharge | No |
| 11 | No | No | No | Good tolerance | Other | Alive at discharge | No |
| 12 | No | No | No | Good tolerance | PPHN | Deceased | No |
| 13 | No | No | No | Good tolerance | PPHN | Deceased | Decrease in MAP and oxygenation |
| 14 | No | No | No | Good tolerance | PPHN | Deceased | Decrease in MAP alone |
| 15 | No | No | No | Good tolerance | PPHN | Deceased | Lactate increase alone |
| 16 | No | Yes (-20%) | No | Good tolerance | PPHN | Deceased | No |
| 17 | No | No | No | Good tolerance | PPHN | Deceased | No |
| 18 | No | No | No | Good tolerance | Other | Deceased | No |

Legend: CAC, calibrated abdominal compression; CI, cardiac index; HR, heart rate; MAP, mean systemic arterial pressure; PPHN, persistent pulmonary hypertension of the newborn; VE volume expansion

### Supplementary Table 2. Diagnostic accuracy of ΔSV-AC to assess fluid responsiveness in homogenized subgroups (sensitivity analysis).

| Population | AUROC  [95%CI] | Best cut-off value (%) | Youden index | Sensitivity  [95%CI] | Specificity  [95%CI] | PPV  [95%CI] | NPV  [95%CI] |
| --- | --- | --- | --- | --- | --- | --- | --- |
| Only 10% Albumin VE (*n=13*) | 0.67 [0.32–1] | 29% | 0.50 | 0.50 [0.12–0.88] | 1 [0.59–1] | 1 [0.29–1] | 0.70 [0.35–0.93] |
| Only PPHN/obstructive shock (*n=14*) | 0.82 [0.56–1] | 17% | 0.57 | 0.71 [0.29–0.96] | 0.86 [0.42–1] | 0.83 [0.36–1] | 0.75 [0.35 –0.97] |
| Only patients ventilated in HFO mode (*n=17*) | 0.80 [0.54–1] | 17% | 0.61 | 0.71 [0.29–0.96] | 0.90 [0.55–1] | 0.83 [0.36–1] | 0.82 [0.48–0.98] |
| Only first VE for each patient (*n=10*) | 0.71 [0.35–1] | 3% | 0.50 | 1 [0.40–1] | 0.50 [0.12–0.88] | 0.57 [0.18–0.90] | 1 [0.29–1] |

Legend: 95%CI, 95% confidence interval; AUROC, area under the receiving operative curve; HFO, high frequency oscillation; NPV, negative predictive value; PPHN, pulmonary hypertension of the newborn; PPV, positive predictive value; VE volume expansion; ΔSV-AC, percentage of indexed stroke volume variation between baseline and during calibrated abdominal compression.

### Supplementary Table 3. Diagnostic accuracy of ΔSV-AC using alternative definitions of fluid responsiveness (sensitivity analysis)

| Fluid responsiveness definition | AUROC  [95%CI] | Best cut-off value (%) | Youden index | Sensitivity  [95%CI] | Specificity  [95%CI] | PPV  [95%CI] | NPV  [95%CI] |
| --- | --- | --- | --- | --- | --- | --- | --- |
| *Increase in cardiac index* |  |  |  |  |  |  |  |
| 10% | 0.83 [0.64–1] | 12% | 0.64 | 0.64 [0.31–0.89] | 1 [0.59–1] | 1 [0.59–1] | 0.64 [0.31–0.89] |
| 15% | 0.73 [0.48–0.98] | 17% | 0.44 | 0.56 [0.21–0.86] | 0.89 [0.52–1] | 0.83 [0.36–1] | 0.67 [0.35–0.90] |
| 20% | 0.76 [0.45–1] | 28% | 0.67 | 0.67 [0.22–0.96] | 1 [0.74–1] | 1 [0.40–1] | 0.86 [0.57–0.98] |
| *Increase in SVi* |  |  |  |  |  |  |  |
| 10% | 0.84 [0.63–1] | 7% | 0.68 | 0.80 [0.44–0.97] | 0.88 [0.47–1] | 0.89 [0.52–1] | 0.78 [0.40–0.97] |
| 15% | 0.79 [0.55–1] | 17% | 0.52 | 0.63 [0.24–0.91] | 0.90 [0.55–1] | 0.83 [0.36–1] | 0.75 [0.43–0.95] |
| 20% | 0.79 [0.52–1] | 17% | 0.62 | 0.71 [0.29–0.96] | 0.91 [0.59–1] | 0.83 [0.36–1] | 0.83 [0.52–0.98] |

Legend: 95%CI, 95% confidence interval; AUROC, area under the receiving operative curve; NPV, negative predictive value; PPV, positive predictive value; SVi, indexed stroke volume; ΔSV-AC, percentage of indexed stroke volume variation between baseline and during calibrated abdominal compression.

### Supplementary Table 4. Baseline parameters associated with fluid responsiveness: univariable binomial logistic regression.

| **Variable** | **Odds Ratio** | **95%CI** | ***p* value** |
| --- | --- | --- | --- |
|  |  |  |  |
| Weight > 750g | 1.67 | 0.25–11.07 | 0.597 |
| Female sex | 5.40 | 0.44–66.67 | 0.188 |
| Birth gestational age > 26 weeks | 6.67 | 0.81–54.96 | 0.078 |
| Adjusted gestational age > 28 weeks | 1.50 | 0.23–9.80 | 0.672 |
| SpO2/FiO2 ratio < 150 | 2.00 | 0.26–15.38 | 0.505 |
| OSI (+1) | 1.01 | 0.85–1.20 | 0.896 |
| PPHN-related shock | 3.00 | 0.25–36.32 | 0.388 |
| VIS > 30 | 1.40 | 0.20–10.03 | 0.738 |
| Heart rate > 180.min^-1^ | 2.40 | 0.29–19.78 | 0.416 |
| Mean arterial pressure < 40mmHg | 0.40 | 0.06–2.70 | 0.347 |
| Urine output (+1mL.kg^-1^.h^-1^) | 1.27 | 0.90–1.78 | 0.172 |
| Lactate > 2 mmol.L^-1^ | 4.67 | 0.40–53.95 | 0.217 |
| LVEF (+1%) | 0.91 | 0.79–1.04 | 0.148 |
| **VTI (+1cm)** | **0.43** | **0.19–0.99** | **0.047** |
| MAFV (+1 cm.s^-1^) | 0.83 | 0.68–1.01 | 0.067 |
| Cardiac index < 200ml.kg^-1^.min^-1^ | 9.00 | 0.75–108.31 | 0.083 |

Legend: FiO2, fraction of inspired oxygen; LVEF, left ventricular ejection fraction; MAFV, mean aortic flow velocity; OSI, oxygen saturation index = mean airway pressure (cmH2O) x FiO2/SpO2; PPHN, persistent pulmonary hypertension of the newborn; SpO2, peripheral oxygen saturation; VE, volume expansion; VIS, vasoactive inotropic score; VTI, aortic velocity-time integral. Significant *p* values are marked in bold.

### Supplementary Table 5. Baseline parameters associated with fluid responsiveness: univariable binomial logistic regression adjusted for repeated measurements within patients.

| **Variable** | **Odds Ratio** | **95%CI** | ***p* value** |
| --- | --- | --- | --- |
|  |  |  |  |
| Weight > 750g | 1.50 | 0.26–8.55 | 0.649 |
| Female sex | 4.90 | 0.25–95.78 | 0.295 |
| Birth gestational age > 26 weeks | 8.10 | 0.55–118.7 | 0.127 |
| Adjusted gestational age > 28 weeks | 1.22 | 0.13–11.71 | 0.862 |
| SpO2/FiO2 ratio < 150 | 1.35 | 0.18–9.95 | 0.766 |
| OSI (+1) | 0.97 | 0.83–1.12 | 0.665 |
| PPHN-related shock | 2.88 | 0.21–39.32 | 0.427 |
| VIS > 30 | 1.13 | 0.26–4.90 | 0.869 |
| Heart rate > 180.min^-1^ | 1.86 | 0.40–8.67 | 0.427 |
| Mean arterial pressure < 40mmHg | 0.39 | 0.06–2.65 | 0.337 |
| Urine output (+1mL.kg^-1^.h^-1^) | 1.24 | 0.91–1.69 | 0.172 |
| Lactate > 2 mmol.L^-1^ | 4.36 | 0.62–30.78 | 0.140 |
| LVEF (+1%) | 0.89 | 0.76–1.04 | 0.15 |
| **VTI (+1cm)** | **0.42** | **0.25–0.68** | **<0.001** |
| **MAFV (+1 cm.s^-1^)** | **0.83** | **0.70–0.99** | **0.042** |
| Cardiac index < 200ml.kg^-1^.min^-1^ | 7.17 | 0.94–54.54 | 0.057 |

Legend: FiO2, fraction of inspired oxygen; LVEF, left ventricular ejection fraction; MAFV, mean aortic flow velocity; OSI, oxygen saturation index = mean airway pressure (cmH2O) x FiO2/SpO2; PPHN, persistent pulmonary hypertension of the newborn; SpO2, peripheral oxygen saturation; VE, volume expansion; VIS, vasoactive inotropic score; VTI, aortic velocity-time integral. Significant *p* values are marked in bold. All analyses were adjusted for repeated measurements within patients using generalized estimating equations.
